# Left-Side Pressure Index for All-Cause Mortality in Older Adults with HFpEF: Diagnostic Potential for HFpEF and Possible View for HFrEF

**DOI:** 10.3390/jcm12030802

**Published:** 2023-01-19

**Authors:** Shiro Hoshida

**Affiliations:** Department of Cardiovascular Medicine, Yao Municipal Hospital, 1-3-1 Ryuge-cho, Yao 581-0069, Osaka, Japan; shiro.hoshida@hosp-yao.osaka.jp; Tel.: +81-72-922-0881; Fax: +81-72-924-4820

## Abstract

Heart failure (HF) with preserved ejection fraction (HFpEF) is thought to be driven by increased cardiac afterload, which consequentially leads to left ventricular (LV) diastolic dysfunction. The ratio of LV diastolic elastance (Ed) to arterial elastance (Ea) significantly increases in older hypertensive women without HF and is coincident with cardiac structural alterations. Ed/Ea is reported to be a prognostic factor for all-cause mortality in patients admitted with HFpEF. In this short article, I provide a possible view of this novel index as having diagnostic potential for HFpEF in clinics and playing a prognostic role in HF with reduced ejection fraction (HFrEF).

Heart failure (HF) with preserved ejection fraction (HFpEF) is associated with increased hospitalizations, especially within the older population. After an older patient admitted with HFpEF is discharged, medical therapy aims to reduce the likelihood of their re-admission for HF and to ease the socioeconomic burden on healthcare. However, considering that Japan is expecting an increase in healthy life expectancy stemming from its super-aging society and declining population, it is important to reduce both re-admission for HF and all-cause mortality in ordinary people.

Historically, HFpEF was thought to be driven by increased cardiac afterload, which consequentially was taken to lead to left ventricular (LV) diastolic dysfunction. There were no pharmacological agents that could reduce all-cause mortality in patients with HFpEF; sodium-glucose co-transporter 2 inhibitors showed no advantage in reducing all-cause mortality [1,2], even in a usual meta-analysis [3]. However, a utility with this agent in mortality was reported recently in a patient-level pooled meta-analysis [4] and a comprehensive meta-analysis of five randomized controlled trials [5]. Although no hemodynamic indices related to all-cause mortality were reported for patients with HFpEF, we recently found that the ratio of operant LV diastolic elastance (Ed) to effective arterial elastance (Ea) [Ed/Ea = ratio of E/e’ to 0.9 × systolic blood pressure] significantly increased in older (age ≥ 75 years) hypertensive women without HF admission and was coincident with cardiac structural alterations [6]. This novel index reflects left atrial pressure relative to systemic pressure [7] (ratio of left atrial filling pressure to LV end-systolic pressure) [8] (Figure 1). Ed/Ea could be an alternative diagnostic tool for HFpEF in outpatient clinics owing to the difficulty of diagnosing HFpEF in patients without HF admission in a clinical setting.

To determine the prognostic factors in patients with HFpEF, important clinical variables including laboratory data such as N-terminal pro B-type natriuretic peptide (NT-proBNP) and evidence of comorbidities must be adjusted in addition to age and sex during multivariable model analysis. Under these conditions, Ed/Ea is a prognostic factor for all-cause mortality in addition to re-admission for HF in patients with HFpEF [9]. This issue clearly demonstrates that the etiology of the occurrence of all-cause mortality or re-admission for HF is ultimately related to the reduction in left-side cardiac function, even if LV ejection fraction is preserved. The prognostic capacity of the Ed/Ea ratio in patients with HFpEF is superior to that of other hemodynamic prognostic factors, such as E/e’ [9]. In HFpEF patients with a higher level of NT-proBNP, higher Ed/Ea was associated with a poor prognosis [10]. The clinical significance of prognostic factors related to hemodynamics may differ based on follow-up period and sex [8]. As Ed/Ea alters according to changes in hemodynamic state, serial measurement of Ed/Ea is needed to accurately diagnose HFpEF prognosis.

It is unknown whether Ed/Ea level directly results in a poor prognosis in patients with HF with reduced ejection fraction (HFrEF). Reduced LV ejection fraction could improve to the normal range in cases of reversible ischemia or diabetic origin. Under these conditions, the Ed/Ea levels would change in accordance with changes in pathophysiological status on a moment-to-moment basis. By serially examining changes in Ed/Ea levels, one may reveal that the patients with reversible HFrEF could have a better prognosis. For example, the reversal of myocardial ischemia would cause the elevation of LV systolic function, leading to improved systolic blood pressure. Furthermore, this would recover LV diastolic function, which in turn could change depending on the severity of myocardial ischemia. When recovering from myocardial ischemia, the improvement of diastolic function requires a longer duration than that of systolic function; [11] thus, the change could be detected easier when monitoring LV systolic function in a clinical setting. It is a future clinical concern whether serial measurement of Ed/Ea level could evaluate cardiac pathophysiological changes and thus establish prognostic significance in patients with HFrEF as in those with HFpEF.

In conclusion, Ed/Ea, a prognostic factor for all-cause mortality in HFpEF, has a possible role to play in diagnosing HFpEF in clinics and may have an alternative significance in manipulating the patients with HFrEF.

## Figures and Tables

**Figure 1:**
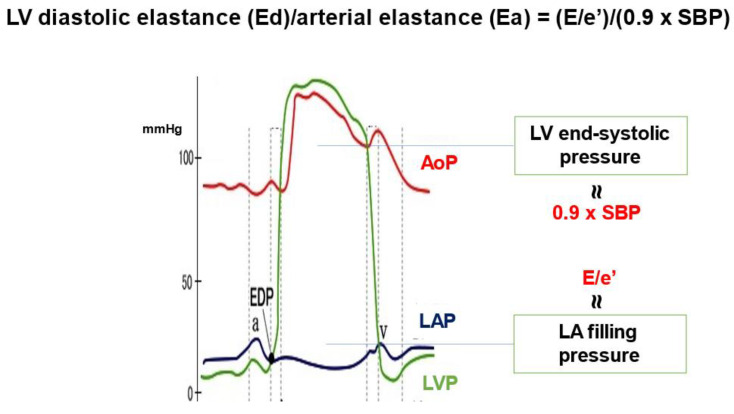
Ratio of left ventricular (LV) diastolic elastance (Ed) to arterial elastance (Ea) nearly represents that of left atrial (LA) filling pressure to LV end-systolic pressure. AoP, aortic pressure; EDP, end-diastolic pressure; LAP, left atrial pressure; LVP, left ventricular pressure; SBP, systolic blood pressure.

## Data Availability

Not applicable.
